# Why Should the “Alternative” Method of Estimating Local Interfacial Shear Strength in a Pull-Out Test Be Preferred to Other Methods?

**DOI:** 10.3390/ma11122406

**Published:** 2018-11-28

**Authors:** Serge Zhandarov, Edith Mäder, Uwe Gohs

**Affiliations:** 1 Leibniz-Institut für Polymerforschung Dresden e.V., Hohe Str. 6, D-01069 Dresden, Germany; emaeder@ipfdd.de (E.M.); gohs@ipfdd.de (U.G.); 2 “V.A.Bely” Metal-Polymer Research Institute, National Academy of Sciences of Belarus, Kirov Str. 32a, 246050 Gomel, Belarus

**Keywords:** pull-out test, local interfacial shear strength, interfacial frictional stress, analysis of force–displacement curves, debond force, “alternative” method

## Abstract

One of the most popular micromechanical techniques of determining the local interfacial shear strength (local IFSS, *τ_d_*) between a fiber and a matrix is the single fiber pull-out test. The *τ_d_* values are calculated from the characteristic forces determined from the experimental force–displacement curves using a model which relates their values to local interfacial strength parameters. Traditionally, the local IFSS is estimated from the debond force, *F_d_*, which corresponds to the crack initiation and manifests itself by a “kink” in the force–displacement curve. However, for some specimens the kink point is hardly discernible, and the “alternative” method based on the post-debonding force, *F_b_*, and the maximum force reached in the test, *F*_max_, has been proposed. Since the experimental force–displacement curve includes three characteristic points in which the relationship between the current values of the applied load and the crack length is reliably established, and, at the same time, it is fully determined by only two interfacial parameters, *τ_d_* and the interfacial frictional stress, *τ_f_*, several methods for the determination of *τ_d_* and *τ_f_* can be proposed. In this paper, we analyzed several theoretical and experimental force–displacement curves for different fiber-reinforced materials (thermoset, thermoplastic and concrete) and compared all seven possible methods of *τ_d_* and *τ_f_* calculation. It was shown that the “alternative” method was the most accurate and reliable one, while the traditional approach often yielded the worst results. Therefore, we proposed that the “alternative” method should be preferred for the experimental force–displacement curves analysis.

## 1. Introduction

The single fiber pull-out test [1,2,3,4] is probably the most popular micromechanical technique for determining the interfacial strength parameters in fiber–matrix systems. Since its invention in the early 60s [1], this technique has been greatly improved and further developed concerning both its experimental part and the data reduction. For a long time, the quality of interfacial bonding was characterized in terms of the apparent interfacial shear strength (apparent IFSS, *τ_app_*) defined as [5,6].

(1) $$\tau_{app}=\frac{F_{max}}{\pi d_{f}l_{e}}$$

where *F*_max_ is the maximum force registered in the pull-out test, *d_f_* is the fiber diameter and *l_e_* is the embedded fiber length.

**Figure 1:**
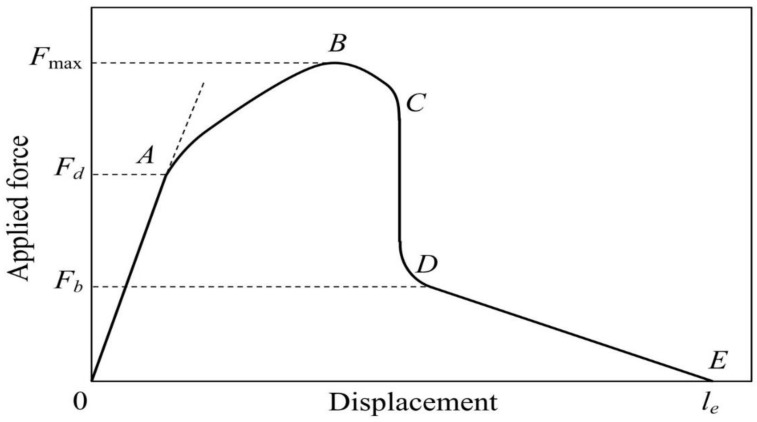
An idealized force–displacement curve in the pull-out test (for details, see Introduction).

This approach is experimentally very simple, and the calculation of *τ_app_* requires the knowledge of the fiber diameter, embedded length and the force required for complete fiber pull-out. The *τ_app_* value calculated using Equation (1) was often referred to as “interfacial adhesion”, “adhesive strength” or “bond strength” [1,7,8]. Much later came the understanding that to the apparent IFSS contributes, except adhesion, also interfacial friction between the fiber and the matrix [9,10]. This is due to the mechanism of interfacial debonding. It was shown both theoretically [10,11,12,13] and experimentally [13,14,15,16,17,18,19] that it occurs through interfacial crack propagation (unzipping). The effects of adhesion and friction can be understood when we consider a force–displacement curve recorded during the pull-out test (Figure 1). It can be divided into several consecutive segments. At the first stage (*OA*, initial loading), the interface is intact and fiber end displacement is nearly proportional to the applied force. At point *A*, the force becomes sufficient to initiate interfacial debonding (*F* = *F_d_*, “debond force”). From this moment, friction in debonded regions begins to contribute to the current force value. At point *B*, the intact interface region becomes too short, and further crack propagation only can decrease the force, in spite of continuously increasing crack length. Therefore, the recorded force shows its maximum value there (*F* = *F*_max_). Further, debonding becomes instable at point *C.* Consequently, the remaining embedded area instantly debonds and the force dropped to a smaller value, *F_b_*. The remaining segment, *DE*, is due to frictional interaction during pull-out of the debonded fiber; the force here is nearly proportional to the length of the fiber part remaining within the specimen. Note that the length of this segment (*DE*) is presented in Figure 1 not to scale, in order to explicitly show point *E* which is used during the experimental data treatment to determine the embedded fiber length (*OE* = *l_e_*). In real pull-out experiments, debonding typically gets completed (point *D*) at the displacements of less than 20–25 µm, while the embedded length can be much greater (up to several millimeters).

Analysis of experimental force–displacement curves promoted a change from the averaged (apparent) *τ_app_* value to local interfacial parameters, which can be considered as debonding criteria and “true” characteristics of the interface strength. Two large groups of theoretical models based on two different debonding criteria have been developed. In stress-controlled debonding models [4,5,20,21,22,23,24], the local (ultimate) interfacial shear strength, *τ_d_*, considered as the local shear stress near the crack tip, is supposed to be constant during the test (and thus independent of the crack length, *a*). Models of energy-controlled debonding [10,12,25,26,27,28] assume that the interfacial crack is initiated when the energy release rate, *G*, reaches its critical value, *G_ic_*, and further crack propagation proceeds at constant *G* value (*G* = *G_ic_*). In this approach, the critical energy release rate can also be considered as local interfacial strength parameter, called also interfacial toughness. It was shown that the local IFSS and the critical energy release rate are practically equivalent as criteria for interfacial crack initiation (the onset of debonding) and, moreover, the experimental force–displacement curves from the pull-out test can be successfully modeled using both energy-base and stress-base approaches [23]. In this paper, we will limit to the stress-based approach, with the local interfacial strength, *τ_d_*, as debonding criterion.

Both stress-based and energy-based approaches relate the appropriate local interfacial strength parameter (*τ_d_* or *G_ic_*) to the debond force, *F_d_*, which manifests itself as a “kink” in the force–displacement curve [12,13,19,23]. Therefore, *F_d_* becomes the most important experimental quantity which should be determined as accurately as possible. Modern installations for pull-out testing [29,30,31,32,33,34] are much more sophisticated than old devices whose only task was to measure *F*_max_ in order to further calculate *τ_app_*. The fiber is pulled out from the matrix with a small controlled speed (displacement rate); geometrical dimensions of the matrix droplet required for *τ_d_* calculation are accurately determined; the fiber diameter is measured in a strong optical or electron microscope with an accuracy of 0.01 µm. As a result, a very accurate force–displacement curve is recorded whose general shape is similar to that shown in Figure 1. It may seem that getting the *F_d_* value from this curve and subsequent local IFSS calculation using one of available stress-based debonding models should be a rather simple task.

Unfortunately, in some cases the debond force value cannot be reliably determined from the force–displacement curve. If the test equipment is not stiff enough (e.g., when the free fiber length between the matrix droplet top and the fixed opposite fiber end is too large), the curve slope changes at point *A* only slightly, so that the kink corresponding to the debonding onset is not discernible [12,13,19,35,36]. For some fiber–matrix pairs, plasticity of the matrix may be responsible for the first “kink” in the force–displacement curve, especially if the local IFSS is close to the matrix shear strength [37]. For some other systems, even force–displacement curves obtained using stiff pull-out installations show no kink, or there can be multiple kinks in the curve, and the *F_d_* value cannot be determined reliably [34]. The examples of such force–displacement curves and the discussion of possible reasons for this behavior are given below in Section 5.

To avoid these problems, we proposed a method for local IFSS determination based on other characteristic points of force–displacement curves (*F*_max_ and *F_b_*), without using the *F_d_* value (“alternative method”) [38,39]. Research over the last few years has put forth evidence that this method successfully works for many fiber–matrix systems and is often more reliable than the traditional method using *F_d_* [40,41]. The further sections of this paper plan to:

- briefly present the model and main equations used to calculate the local interfacial strength parameters from a recorded force–displacement curve;
- show how different methods for *τ_d_* determination can be developed using different sets of characteristic points;
- estimate the accuracy and “general quality” of all these methods by applying them to determine the local IFSS and the interfacial frictional stress, *τ_f_*, from theoretical and experimental (for various fiber–matrix pairs) force–displacement curves;
- discuss the problems encountered in estimating *τ_d_* and *τ_f_* from force–displacement curves for different systems and under different conditions, and recommend the most reliable method if possible.

## 2. The Model

In this paper, we will follow our own stress-based model first introduced in [23] and then successfully used to analyze interfacial strength properties for various combinations of fibers and matrices. It is based on the one-dimensional shear-lag method proposed for fiber–matrix systems by Cox [42] but using corrected shear-lag parameter proposed by Nayfeh [43]. The model includes interfacial friction and thermal shrinkage characteristic for systems with polymer matrices. We make the usual assumptions for such kind of models: (1) both the fiber and the matrix can be considered as perfectly elastic; the matrix is isotropic and the fiber is transversely isotropic; (2) the matrix droplet is considered as a cylinder whose radius is equal to the total specimen volume within the embedded fiber region (“equivalent cylinder” [12,36]), and the fiber is also cylindrical and is embedded in the matrix co-axially; and (3) the interfacial frictional stress in the debonded regions, *τ_f_*, is constant during the test [12,44]. Detailed analysis of the pull-out test based on this model can be found in [23,24,35,38]. For our further consideration, it is very important that the model gives direct expression for the current force, *F*, applied to the fiber, as a function of the crack length, *a* [23]:

(2) $$F\left(a\right)=\frac{2\pi r_{f}}{\beta }\left\{\tau_{d}\tanh\left[\beta \left(l_{e}-a\right)\right]-\tau_{T}\tanh\left[\beta \left(l_{e}-a\right)\right]\tanh\frac{\tau_{d}\tanh\left[\beta \left(l_{e}-a\right)\right]}{2}+\beta a\tau_{f}\right\}$$

where *r_f_* = *d_f_*/2 is the fiber radius; *β* is the shear-lag parameter as defined by Nayfeh [43]

(3) $$\beta^{2}=\frac{2}{r_{f}^{2}E_{A}E_{m}}\left[\frac{E_{A}V_{f}+E_{m}V_{m}}{\frac{V_{m}}{4G_{A}}+\frac{1}{2G_{m}}\left(\frac{1}{V_{m}}\ln\frac{1}{V_{f}}-1-\frac{V_{f}}{2}\right)}\right]$$

and *τ_T_* is a term having dimensions of stress, which appears due to residual thermal stresses [23,35]:

(4) $$\tau_{T}=\frac{\beta r_{f}E_{A}}{2}\left(\alpha_{A}-\alpha_{m}\right)\Delta T$$

In Equations (3) and (4), *E_A_* and *E_m_* are the axial tensile modulus of the fiber and the tensile modulus of the matrix, respectively, *V_f_* and *V_m_* are the fiber and matrix volume fractions within the specimen, *G_A_* and *G_m_* are the axial shear modulus of the fiber and the shear modulus of the matrix, *α_A_* is the axial coefficient of thermal expansion (CTE) of the fiber, *α_m_* is the CTE of the matrix, and ∆*T* is the difference between the test temperature and a stress-free temperature which, for polymers, is usually assumed to be equal to the glass transition temperature (or to the room temperature, if it is above the glass transition temperature).

As can be seen, the *F*(*a*) value depends, except the crack length, on the fiber and matrix properties, specimen geometry and two interfacial parameters, *τ_d_* and *τ_f_*. In the force–displacement curve (see Figure 1), the whole region corresponding to the crack propagation (from *a* = 0 to *a* = *l_e_*) is represented by segment *ABCD*, which includes all three characteristic points (*A*, *B* and *D*). Thus, we can write Equation (2) for these points and then consider resulting equations as implicit equations for *τ_d_* and *τ_f_*.

Point *A* (*a* = 0, *F* = *F_d_*):

(5) $$F_{d}=\frac{2\pi r_{f}}{\beta }\left[\tau_{d}\tanh\left(\beta l_{e}\right)-\tau_{T}\tanh\left(\beta l_{e}\right)\tanh\left(\frac{\beta l_{e}}{2}\right)\right]$$

Point *D* (*a* = *l_e_*, *F* = *F_b_*):

(6) $$F_{b}=2\pi r_{f}a\tau_{f}$$

Point *B* (*F* = *F*_max_). The equation for *F*_max_ cannot be derived so easily as for *F_d_* and *F_b_*, since the crack length at point *B* is a priori unknown. Nevertheless, we have found its explicit form [24]:

(7) $$F_{\max}=\{\begin{array}{l} \frac{2\pi r_{f}}{\beta }\left[\tau_{d}\tanh\left(\beta l_{e}\right)-\tau_{T}\tanh\left(\beta l_{e}\right)\tanh\left(\frac{\beta l_{e}}{2}\right)\right],\;\;\beta l_{e}<\omega ;\\ \frac{2\pi r_{f}}{\beta }\left\{\tau_{d}\frac{u}{\sqrt{u^{2}+1}}-\tau_{T}\left(1-\frac{1}{\sqrt{u^{2}+1}}\right)+\tau_{f}\left(\beta l_{e}-\omega \right)\right\},\;\beta l_{e}\geq \omega , \end{array}$$

where

(8) $$u=\frac{\sqrt{\tau_{T}^{2}+4\tau_{f}\left(\tau_{d}-\tau_{f}\right)}-\tau_{T}}{2\tau_{f}}$$

(9) $$\omega =\ln\left(u+\sqrt{u^{2}+1}\right)$$

We have three implicit Equations (5)–(7) for two unknown variables, *τ_d_* and *τ_f_*; all others variables and constants in these equations are known. So, we can choose several different methods to solve this overdetermined set of equations. This will be discussed in Section 3.

All calculations for this paper were performed in the programming environment Mathematica 10.3 by Wolfram Research, Inc. (Champaign, IL, USA) [45].

## 3. Methods for Determination of Interfacial Strength Parameters

In this Section, we will present possible methods for determination of the interfacial strength parameters, *τ_d_* and *τ_f_*. We should note that each method must show a way to calculate both parameters; in other words, methods differing in algorithms of determination of at least one parameter are considered to be different.

It is easy to see that the local interfacial shear strength, *τ_d_*, can immediately be determined from Equation (5) without considering other equations of the set:

(10) $$\tau_{d}=\frac{F_{d}\beta }{2\pi r_{f}}\coth\left(\beta l_{e}\right)+\tau_{T}\tanh\left(\frac{\beta l_{e}}{2}\right)$$

This is the basis for the “traditional” approach to the *τ_d_* determination (from the debond force value). At the same time, the frictional stress, *τ_f_*, can be found in three different ways. This yields the first three methods for *τ_d_* and *τ_f_* determination.

Method 1 (“traditional”). The local IFSS is calculated using Equation (10). Then this value is substituted into Equation (7), and the resulting implicit equation is solved for *τ_f_*. This method was widely used in our work [23,24,35,36,46,47] before we have developed the “alternative” method. In most experimental papers in the literature, e.g., [48,49,50,51,52,53,54], researchers were not interested in the *τ_f_* value but calculated *τ_d_* from the debond force, using Equation (10) or a similar one. These papers we will also conditionally refer to as using the “traditional” approach.

**Figure 2:**
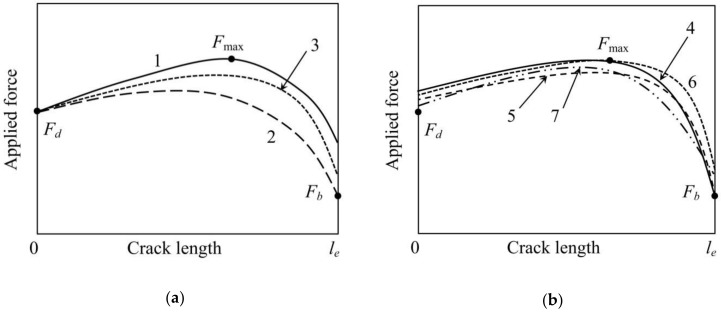
Schematic force–crack length curves illustrating the methods used for determination of interfacial strength parameters: (**a**), methods directly based on the debond force, *F_d_*; (**b**), all other methods. The curve numbers correspond to the numbers of methods as listed in Section 3.

In this method, Equation (6) is not used. It is interesting to substitute into it the calculated *τ_f_* value and compare the resulting *F_b_* to its experimental value. If the investigated specimen satisfied all assumptions made in Section 2 (ideally cylindrical shape, absolute elasticity, constant interfacial friction), the calculated and experimental *F_b_* values should be equal. In practice, however, the calculated *F_b_* value is somewhat greater than the experimental one. This is shown in Figure 2a (curve 1) which schematically presents force–crack length curves for different methods considered.

Method 2. This method was proposed quite recently by Textechno Herbert Stein GmbH & Co. KG [55] and implemented in the commercial fiber–matrix adhesion tester FIMATEST [31]. The *F_d_* value is used to determine *τ_d_* from Equation (10), and the interfacial frictional stress, *τ_f_*, is calculated using Equation (6). In some sense, this is a kind of “hybrid” of the “traditional” and “alternative” methods (the latter is discussed below under “Method 4”). The *F*_max_ value is ignored in this method; it is regrettable, since *F*_max_ is measured with the best accuracy of all forces in the characteristic points. The calculated *F*_max_ values for most systems are smaller than the experimental ones (Figure 2a, curve 2).

Method 3. The local IFSS is calculated using Equation (10), as in the two previous methods. However, the *τ_f_* value is determined from a statistical consideration: the force–crack length curve should be “the best” one, i.e., provide the minimum sum of the least squares

(11) $$s_{3}={\left(F_{\max}-F_{3\max}\right)}^{2}+{\left(F_{b}-F_{3b}\right)}^{2}$$

where *F*_max_ and *F_b_* are experimental values, and *F*_3max_ and *F*_3*b*_ are theoretical values satisfying set of Equations (6) and (7). The *s*_3_ minimization can be carried out using the interval bisection method, starting (for *τ_f_*) from the interval (0, *τ_d_*). The best curve and corresponding values of all forces are shown in Figure 2a, curve 3.

Note that we can also formally calculate the minimum sum of the least squares for previous methods. For Method 1, $s_{1}={\left(F_{b}-F_{1b}\right)}^{2}$; for Method 2, $s_{2}={\left(F_{\max}-F_{2\max}\right)}^{2}$.

Each of Methods 1–3 could be successfully used for determination of interfacial strength parameters if there were no problems with accurate *F_d_* determination from experimental force–displacement curves. The possible error in *F_d_* entails an error in *τ_d_*, which, in turn, results in incorrect *τ_f_* value. Therefore, we should try a method which does not use *F_d_* values.

Method 4 (“alternative”). The interfacial frictional stress, *τ_f_*, is calculated using Equation (6); then this value is substituted into Equation (7), and the resulting implicit equation is solved for *τ_d_*. This method was proposed by Zhandarov and Mäder [38] and then used for the estimation of the interfacial strength parameters in several subsequent papers [39,40,41], some of which also included the energy-based consideration (*G_ic_* and *τ_f_*) [39,41]. The comparison with the traditional method for several fiber–matrix systems showed that *τ_d_* (and *F_d_*) values were similar or slightly greater for the alternative method. It is schematically shown in Figure 2b, curve 4. Since the *F_d_* value is not used, the minimum “sum” is $s_{4}={\left(F_{d}-F_{4d}\right)}^{2}$.

The obvious advantage of this method is that it is based on *F*_max_ and *F_b_* values which can be measured with good accuracy, in contrast to the third characteristic force, *F_d_*.

To complete the picture, we will also present three remaining possible methods for *τ_d_* and *τ_f_* determination which use the characteristic force values in different ways.

Method 5. The *τ_f_* value is calculated from *F_b_* using Equation (6), and *τ_d_* from the minimum sum $s_{5}={\left(F_{d}-F_{5b}\right)}^{2}+{\left(F_{\max}-F_{5\max}\right)}^{2}$ (Equations (5) and (7), curve 5 in Figure 2b).

Method 6. It is based on a force–crack length curve whose maximum coincides with the experimental *F*_max_ point, and the sum $s_{6}={\left(F_{d}-F_{6d}\right)}^{2}+{\left(F_{b}-F_{6b}\right)}^{2}$ reaches its minimum value (curve 6 in Figure 2b). The algorithm of *τ_d_* and *τ_f_* evaluation for this method is more complicated than simple interval bisection used for Methods 3 and 5. First, we should note that *τ_f_* cannot be greater than the apparent IFSS, *τ_app_*. In the interval (0, *τ_app_*) we select a large number (e.g., 1000) *τ_f_* values. Then, for each *τ_f_* value determine the local IFSS (solving the implicit Equation (7) for *τ_d_* using the interval bisection method) and the corresponding sum of the least squares, *s*_6_. The pair {*τ_d_*, *τ_f_*} which yields the least *s*_6_ value is taken as the best estimation of the interfacial strength parameters for this method.

**Figure 3:**
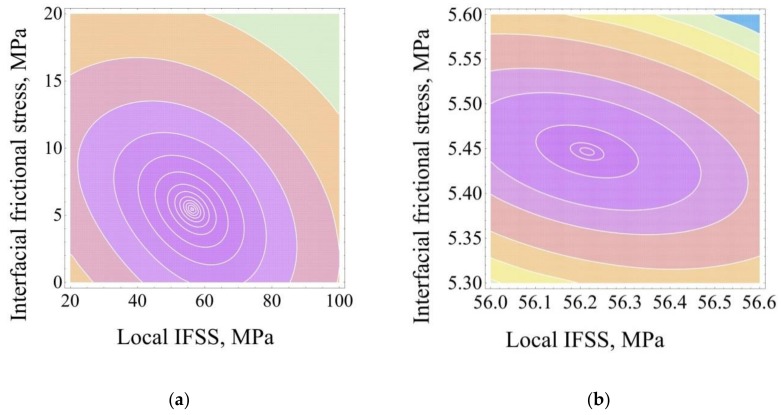
The maps of the sum of least squares for Method 7: (**a**), 1st iteration; (**b**) 3rd iteration. The central point corresponds to the best {*τ_d_*, *τ_f_*} pair.

Method 7. In this method, the best force–crack length curve having form (2) has to minimize the sum $s_{7}={\left(F_{d}-F_{7d}\right)}^{2}+{\left(F_{\max}-F_{7\max}\right)}^{2}+{\left(F_{b}-F_{7b}\right)}^{2}$ (least squares method for all characteristic points, curve 7 in Figure 2b). For Method 7, we calculated *s*_7_ values for many {*τ_d_*, *τ_f_* } pairs falling into the area {0 < *τ_d_* ≤ *τ_d_*_max_, 0 < *τ_f_* ≤ *τ_f_*_max_} (where *τ_d_*_max_ and *τ_f_*_max_ are large enough, e.g., 120–150 MPa for *τ_d_*_max_ and 30–100 MPa for *τ_f_*_max_) and plotted the map of the sum of least squares, *s*_7_, as shown in Figure 3a. Enlarging the scale, it is possible to determine, after 2–3 iterations, the “best” *τ_d_* and *τ_f_* values with good accuracy (Figure 3b). Then these values are used to calculate the best *F*_7*d*_, *F*_7max_ and *F*_7*b*_ values from Equations (5)–(7).

## 4. Evaluation of Interfacial Strength Parameters from Theoretical Force–Displacement Curves: Comparison of the Methods

As already was mentioned above, if all assumptions of the model were satisfied, the theoretical force–crack length curve must go through all three characteristic points, *F_d_*, *F*_max_ and *F_b_*, and not depend on which two points were selected for the evaluation. In other words, all seven above-described methods should result in the same “true” force–crack length curve with the same *τ_d_* and *τ_f_* parameters; all theoretical force–displacement curves also should be identical. However, real experimental curves differ from their ideal shape. The possible reasons can be as follows:

- Non-cylindrical shape of the matrix droplet. The interfacial crack starts at the top of the droplet, where the fiber content is extremely high (well above its mean value, *V_f_*), and then propagates into the regions with continuously decreasing *V_f_*.
- Non-ideal elasticity, especially of the matrix, which distorts the theoretical curve and can affect positions of the characteristic points.
- Too short embedded length; in such specimens, most of the crack may be located in the meniscus region which is essentially non-cylindrical.
- Imperfect interface: large interfacial defects can result in additional “kinks” and decrease the measured debond force.
- Possible movement of the opposite (fixed) fiber end within the glue or in the clamps.
- Non-linear frictional force which indicates substantial effect of transverse (normal) interfacial stresses.

This is only a few of the factors that can affect the shapes of the force–displacement and force–crack length curves. However, the non-cylindrical shape of the specimen is undoubtedly the main factor. In our previous papers [56,57], we investigated crack initiation and propagation within matrix droplets of real shape, i.e., spherical segments with menisci (wetting cones) having different wetting angles in contact with a fiber. We start with these theoretical examples for two reasons: (1) For specimens with a well-defined non-cylindrical shape, we obtained both force–crack length and force–displacement curves, which is typically impossible for real pull-out specimens; and (2) for each theoretical curve, we have pre-set the interfacial parameters, *τ_d_* and *τ_f_*; in other words, we know the “true” values of these parameters, in contrast to real pull-out tests.

**Figure 4:**
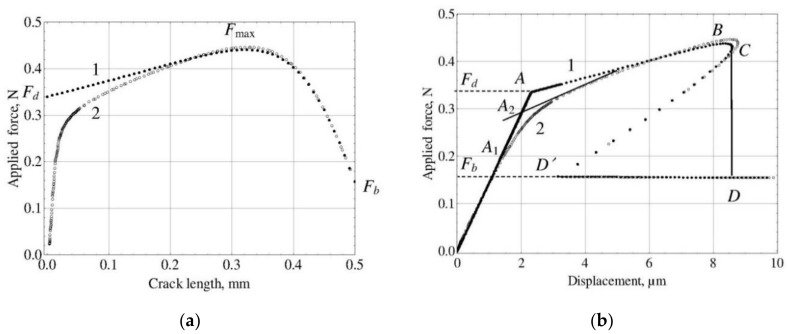
Force–crack length (**a**) and force–displacement (**b**) curves simulated for the glass fiber–epoxy matrix system. The embedded length is 500 µm. Curves 1 correspond to the equivalent cylinder; curves 2, to the real-shaped specimen.

Figure 4 presents the force–crack length (Figure 4a) and force–displacement (Figure 4b) curves simulated for the glass fiber–epoxy matrix system [57]. The mechanical and thermal properties of both components are listed in Table 1. The matrix droplet radius was set to 1.25 mm, which corresponds to the diameter of matrix holder used in our experiments (2.5 mm). The fiber diameter was set to 20 µm, the embedded length, to 500 µm. The wetting angle was 30°, which is typical for fiber–polymer systems [57]. The interfacial strength parameters were set to *τ_d_* = 60 MPa and *τ_f_* = 5 MPa, the free fiber length was assumed to be zero in order to reach maximum stiffness of the virtual “testing installation”. For comparison, the “equivalent cylinder” specimen having the same embedded length and total volume was investigated.

As can be seen in Figure 4a, in the cylindrical specimen interfacial crack starts at a final and rather large applied force value, *F_d_* = 0.3401 N. Then, as the crack propagates, the force continuously increases to its maximum value (*F*_max_ = 0.4412 N at *a* = 0.325 mm) and then drops to the post-debonding value (*F_b_* = 0.1571 N at *a* = *l_e_* = 0.5 mm). The corresponding force–displacement curve is shown in Figure 4b by filled circles. Its shape is typical for fiber pull-out from cylindrical specimens ([23]; cf. also Figure 1). The segment *CD′D* is experimentally unobservable, since the loaded fiber end cannot move in the reverse direction. The kink corresponding to debonding onset at point *A* is very pronounced, and the *F_d_* value can easily be determined “experimentally”. The *τ_d_* value calculated from Equation (10) using *F_d_* = 0.3401 N is 60 MPa as pre-set.

However, both curves for the specimen with the meniscus show quite different behavior. The crack initiates at very small applied force, practically zero, and then propagates very slowly but with steady growing speed as the applied force is increased. Only from *a* ≈ 0.4 *l_e_* = 0.2 mm, the force–crack length force curves for “real” and cylindrical specimens became very similar. The maximum force value for the “real” specimen is reached at *a* = 0.321 mm and is equal to *F*_max_ = 0.4466 N; the post-debonding force, *F_b_*, is equal for both specimens (*F_b_* = 0.1571 N) since it does not depend on crack propagation. However, the character of initial crack propagation in the “real” specimen results in a smooth force–displacement curve (Figure 4b, curve 1) in which the kink is hardly discernible. Its position can be determined only, to a great extent, arbitrarily. One possible choice is to select the point at which the curve begins to deviate from a straight line (*A*_1_); for this point, *F_d_* = 0.2 N. Another choice has been proposed by Textechno [31,55]. In their approach, two tangent lines were drawn at two successive segments of the force–displacement curve, and the *F_d_* value was taken at the point of their intersection (*A*_2_). For this point, *F_d_* = 0.2939 N. Both *F_d_* values obviously result in *τ_d_* underestimation: Equation (10) yields *τ_d_* = 40.53 MPa for *A*_1_ and *τ_d_* = 53.53 MPa for *A*_2_. Since the *F_d_* value calculated for point *A*_2_ is closer to the true local IFSS (60 MPa), the method of kink determination proposed by Textechno should be preferred, in spite of its non-physicality [34]. For our further calculations in which the *F_d_* value is explicitly used, we will take *F_d_* = 0.2939 N.

Table 2 presents the results of determination of the interfacial strength parameters (*τ_d_* and *τ_f_*) using all seven methods presented in Section 3. The “experimental” values of the characteristic force values are shown in the last string of the table. Parameter *s* is the sum of the least squares, and “Rank” was assigned to the methods according to the calculated *s* values (from the least to the greatest). As could be expected, the best *s* value was obtained for the method 7 in which all three characteristic forces (*F_d_*, *F*_max_ and *F_b_*) were chosen as fitting parameters. Methods 5, 3 and 6 with two fitting parameters each received ranks from 2 to 4. And, finally, methods which used only one fitting parameter (1, 2 and 4) were ranked as 5–7. However, this does not mean that Method 7 is the best method for *τ_d_* and *τ_f_* determination. In our opinion, the criterion of the methods evaluation should be based on its accuracy in determining the interfacial strength parameters rather than on indirect statistical considerations. And in this sense, the best method is Method 4 which yields an absolutely accurate value for *τ_f_* and gives, for this specimen, only 1.5% error in *τ_d_*. This can be physically understood if we look at Figure 4a. The *F_b_* and *F*_max_ values for the “real” specimen and the equivalent cylinder are very close, and the very unreliable (and, as was shown above, significantly underestimated) *F_d_* value is not used in this method. The question arises, why are the *F_b_* and *F*_max_ values for specimens with such different shapes so close to each other? First, we should note that the post-debonding frictional force, *F_b_*, does not depend on the specimen shape or the pattern of the crack propagation. And close values for *F*_max_ can be explained, in our opinion, by the fact that the maximum force is reached at rather large crack length, deeply inside the matrix droplet, where the matrix shape is much closer to a cylinder than in the meniscus or at the top of the matrix spherical segment. We can expect that for the specimens with short embedded fiber lengths, when the whole fiber is located at the matrix top, the *F*_max_ values may be different. In order to check this, we simulated the pull-out test on a specimen with the same fiber and matrix materials, wetting angle of 30°, but having embedded length of 50 µm.

**Figure 5:**
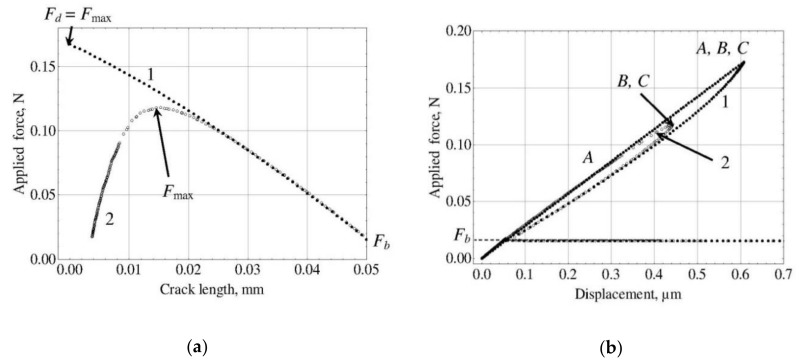
Force–crack length (**a**) and force–displacement (**b**) curves simulated for the glass fiber–epoxy matrix system. The embedded length is 50 µm. Curve 1 corresponds to the equivalent cylinder; curve 2, to the real-shaped specimen.

The force–crack length and force–displacement curves for this specimen are shown in Figure 5. While the force–crack length curve for the “real” specimen is more or less similar to that for 500 µm, for the equivalent cylinder the force steadily decreases from the very crack initiation (Figure 5a), which indicates unstable crack propagation over the whole embedded length. This is also confirmed by the shape of the force–displacement curve (Figure 5b). As can be seen from Figure 5a,b, both *F_d_* and *F*_max_ values for the “real” specimen are considerably lower than those for the equivalent cylinder. This means that the calculated local IFSS (*τ_d_* value) will be underestimated for all seven methods, including Method 4 (since the “experimental” *F*_max_ is also too small!) Nevertheless, Method 4 remains the best method for this specimen with the error in *τ_d_* of “only” 25%. The full results of *τ_d_* and *τ_f_* estimation are presented in Table 3. As can be seen, the methods based on the debond force, *F_d_* (Methods 1–3) yielded the worst *τ_d_* value (23.76 MPa) which is only 39.6% of the true local IFSS.

Thus, we revealed that the embedded length can significantly affect the determined *τ_d_* value. As we found from our practice, the *τ_d_* estimation was satisfactory if *l_e_* > 100…120 µm. In order to be able to test specimens with smaller embedded lengths, we would recommend the use of smaller matrix droplets, for which the specimen shape will be close to cylindrical one. In the next Section, we will consider real (experimental) force–displacement curves obtained by pull-out testing on different fiber–matrix pairs, with different embedded fiber lengths, specimen shapes, etc.

## 5. Evaluation of Interfacial Strength Parameters from Theoretical Force–Displacement Curves: Comparison of the Methods

### 5.1. Experimental

#### 5.1.1. Materials and Specimen Preparation

Properties of fibers and matrices used in our experiments and other data required for *τ_d_* and *τ_f_* calculation are presented in Table 1. We have tested the following fiber–matrix systems: (1) E-glass fibers–Hexion 135 epoxy; (2) Toho Tenax carbon fibers (CF1)–polyamide 6,6 (PA 6,6); (3) Sigrafil C carbon fibers (CF2)–Hexion 135 epoxy; (4) E-glass fibers–polypropylene (PP); and (5) poly(vinyl alcohol) (PVA) fibers–concrete matrix. The conditions of specimen preparation were as follows:

(1) melting for 100 s at 45 °C, then fiber embedding, 1 h at 85 °C and curing for 6 h at 80 °C;
(2) 290 °C/10 s (embedding), then 15 min cooling down to 23 °C;
(3) the same procedure as in (1);
(4) 255 °C/2 min (embedding), then cooling down to 23 °C;
(5) 24 h at 23 °C and RH = 50%, then 1 week at 23 °C and RH = 90%.

#### 5.1.2. Pull-Out Testing

All pull-out tests were carried out at the Leibniz-Institut für Polymerforschung Dresden (IPF) using a specialized apparatus constructed at the IPF and described in detail elsewhere [29]. The pull-out rate was 0.01 µm/s in all cases (quasi-static test). The details of the experimental procedure and data acquisition/initial treatment (recording the force–displacement curve and its further processing in Mathematica) were presented earlier in [38]. For each specimen, three characteristic force values (*F_d_*, *F*_max_ and *F_b_*) were determined from the corresponding force–displacement curve and then used to calculate the interfacial strength parameters (*τ_d_* and *τ_f_*) using all seven methods described in Section 3.

### 5.2. Evaluation Results and Comparison of the Methods

Interfacial strength parameters for real fiber–matrix systems are determined in the same way as for virtual specimens presented in Section 4. However, one important difference is that for real specimens we usually do not have force–crack length curves (unless we specifically investigate the crack propagation, e.g., using Raman spectroscopy [16,17] or direct video recording for transparent matrices [18,19]), and *τ_d_* and *τ_f_* evaluation is based on solely force–displacement curve.

**Figure 6:**
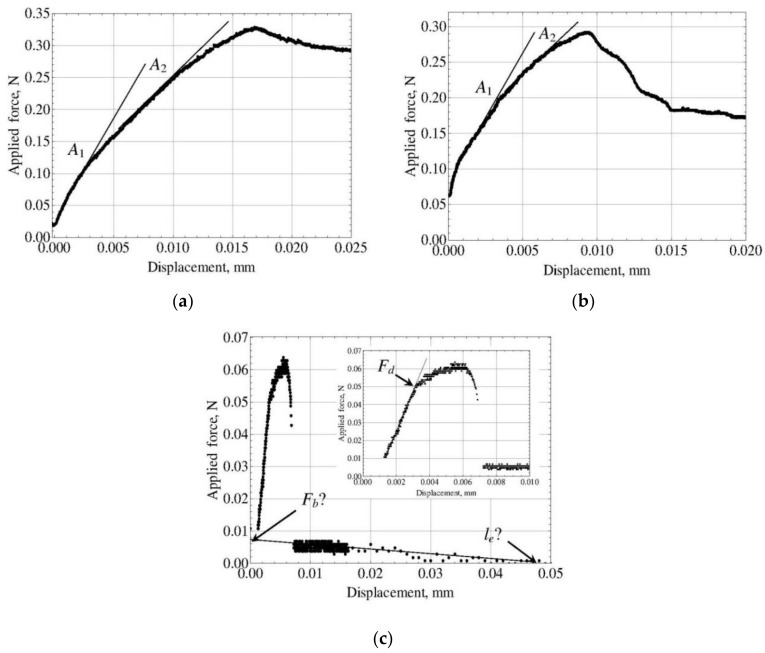
The initial parts of experimental force–displacement curves. (**a**) glass fiber–PP matrix, *l_e_* = 629 µm; (**b**) PVA fiber–concrete matrix, *l_e_* = 1921 µm; (**c**) carbon fiber (CF2)–H135 epoxy matrix, *l_e_* = 47 µm (for this specimen, the full force–displacement curve is also shown).

In the case of glass fiber–PP matrix system, the embedded fiber length in this specimen was 629 µm, so that there were no problems resulting from short embedded length. The general shape of the force–displacement curve for this specimen is rather typical, and the maximum force, *F*_max_, and the post-debonding force, *F_b_*, can easily be determined: *F*_max_ = 0.3296 N and *F_b_* = 0.2867 N (see Table 4). However, two kinks were present in the rising part of the force–displacement curve (Figure 6a). For these kinks, *A*_1_ and *A*_2_ in Figure 6a, we found *F_d_*_1_ = 0.1259 N and *F_d_*_2_ = 0.2659 N, respectively. Then we determined the interfacial parameters, *τ_d_* and *τ_f_*, for both sets of characteristic forces {*F_d_*, *F*_max_, *F_b_*} using all seven methods presented in Section 3. As expected, the pairs {*τ_d_*, *τ_f_*} determined using the “alternative” method (Method 4) appeared to be equal for both sets (17.59 and 8.24 MPa, respectively), since the *F_d_* value is not used in this method. At the same time, all other methods showed a great difference between the results obtained using kink 1 and kink 2. As can be seen in Table 4, the *τ_d_* and *τ_f_* values calculated for kink 1 are close to values determined using Method 4, while for kink 2 all *τ_d_* values are much greater. Obviously, kink 1 is the “true” kink (the corresponding *F_d_* value is in agreement with *F*_max_ and *F_b_* within the frame of the model used), while *F_d_*_2_ = 0.2659 N is highly overestimated. The analysis of the sums of least squares, *s*, confirms this conclusion (see Table 4). Since the embedded fiber length was sufficiently large, we believe that *τ_d_* = 17.59 MPa is close to the “true” local IFSS value. Note that if the kink force is determined wrongly (as for kink 2), the methods which are directly based on the *F_d_* value (Methods 1–3) yield larger errors than the methods which assume that *F_d_* value may be incorrect and combine it with *F_max_* and/or *F_b_* (Methods 5–7).

The PVA fiber–concrete matrix system is very interesting. First, it is one of the few fiber–matrix pairs for which the specimen shape can be considered as very close to cylindrical. Second, large fiber diameters (for the specimen under consideration, *d_f_* = 38.13 µm) and relatively small local IFSS make possible pull-out testing on specimens with very large embedded lengths (*l_e_* = 1921 µm for the considered specimen). Third, the force–displacement curves for this system are more intricate due to slip-dependent friction characteristic of composites with concrete matrices [41,58,59,60,61]. Nevertheless, the maximum force, *F*_max_, and the post-debonding frictional force at the moment of debonding completion, *F_b_*, can be determined reliably using the techniques proposed in [41,58]. For our specimen, we found *F*_max_ = 0.2924 N and *F_b_* = 0.1540 N (Table 5). The initial part of the force–displacement curve also shows two kinks, *A*_1_ and *A*_2_, with corresponding kink forces *F_d_*_1_ = 0.200 N and *F_d_*_2_ = 0.2812 N (Figure 6b). The analysis similar to that described in the previous paragraph shows that the height of point *A*_1_ is only slightly overestimated, while the *τ_d_* values based on *F_d_*_2_ are too large. All conclusions made at the end of the previous paragraph are also valid for the PVA–concrete system. We should only note that overestimated *τ_d_* values for systems with low interfacial friction may often be combined with highly underestimated *τ_f_* values (even by an order of magnitude!)

As can be seen in Section 4, we do not recommend pull-out testing on specimens with very short embedded fiber length. In addition to the above-discussed underestimation of the local IFSS determined by all seven methods, these experiments involve some others, purely technical, problems. Figure 6c presents the force–displacement curve for the carbon fiber (CF2)–H135 epoxy pair. For this type of force–displacement curves, it is rather difficult to draw a straight line corresponding to post-debonding friction. As a result, the *F_b_* value can be determined only very roughly. But the error in the embedded length, *l_e_*, may be much greater, since it is not clear where should the post-debonding straight line cross the displacement axis. For the specimen presented, we determined *F_b_* ≈ 0.00764 N (more or less reliably) and *l_e_* ≈ 47 µm (very rough). Fortunately, this curve includes only one distinct kink in its rising part, so that an approximate estimation of the interfacial parameters can be done. The results are shown in Table 6. Since *τ_d_* = 107 MPa determined using Methods 4 and 6 is greater than *τ_d_* calculated using all other methods, the kink force should be considered as a bit underestimated, which results in a conclusion that the true *τ_d_* value should be even greater. In any case, the test results for such short embedded lengths are very unreliable and can only be used for rough comparative studies. If it is possible, the embedded fiber length in pull-out test should not be less than 100–120 µm.

The force–displacement curves and detailed tables with results for the two remaining fiber–matrix systems (CF1–PA 6,6, *l_e_* = 139 µm and E-glass–H135 epoxy, *l_e_* = 89 µm) are not shown since the general shapes of both curves are regular. The first curve shows one distinct kink; Method 4 yields for this specimen *τ_d_* = 45.80 MPa and *τ_f_* = 7.29 MPa, while other methods which use the *F_d_* estimated *τ_d_* to be between 35 and 42 MPa. This means that the kink position (*F_d_*) is roughly in agreement with the *F*_max_ and *F_b_* values and may be only slightly underestimated, probably due to non-cylindrical shape of the specimen. The agreement between the characteristic force values is also confirmed by very small sums of the least squares, *s*_1_–*s*_7_: all of them are below 0.33 × 10^−3^.

The curve for the E-glass–H135 epoxy includes two kinks in its rising part. Methods using the *F_d_* value yield *τ_d_* = 46…72 MPa (*s* = (30…36) × 10^−3^) for the “lower” kink and *τ_d_* = 85…95 MPa (*s* = (2…4) × 10^−3^) for the “upper” one. Method 4 gave *τ_d_* = 104.8 MPa (and *τ_f_* = 6.43 MPa) for both “kinks”. Obviously, the first kink is “wrong” (is not related to the interfacial crack). At the same time, the second one is, in all probability, rather close to the debond force for the equivalent cylinder, and the value of 104.8 MPa can be considered as a good *τ_d_* estimation for this system.

The factors causing multiple kinks in force–displacement curves still remain, to a great extent, unclear. As can be seen in “theoretical” curves (e.g., Figure 4a), some of the kinks may be artifacts resulting from non-cylindrical specimen shape, even for large embedded lengths. On the other hand, experimental force–displacement curves often show exactly two kinks before reaching the *F*_max_ value; in our opinion, one of these kinks may be due to crack initiation in the glue which holds the opposite fiber end. Since the parameters of the glue droplet (or layer) are typically poorly controlled, the position of this “parasite” kink may vary considerably from one specimen to another. Thus, there is a danger that the wrong kink may be erroneously considered as characterizing the investigated fiber–matrix system. However, if we use several different methods (or at least “traditional” and “alternative” ones) to evaluate an experimental force–displacement curve, the two kinks can be reliably identified, and the “true” one can be chosen.

## 6. Conclusions

We compared seven methods of estimating the local interfacial strength parameters (local IFSS, *τ_d_*, and interfacial frictional stress, *τ_f_*) from force–displacement curves recorded in single fiber pull-out test. All these methods are based on the three characteristic forces which can be determined from the experimental force–displacement curve (debond force, *F_d_*, maximum force, *F*_max_, and initial post-debonding force, *F_b_*) but use these values in different combinations within the frames of a stress-based model of interfacial debonding.

The main reason due to which real experimental force–displacement curves differ from their theoretical shape is non-cylindrical shape of the matrix droplets, especially at their top where the fiber enters the matrix. As a result, the debond force cannot be measured reliably, while the *F*_max_ and *F_b_* values can be determined with good accuracy. Thus, the methods which directly use the debond force, *F_d_*, for *τ_d_* calculation, including the most popular “traditional” method, may yield large errors in the calculated values of the local interfacial strength parameters. Therefore, we propose that the “alternative” method, which does not use *F_d_* at all, should be strongly preferred.

The alternative method yields best results when the embedded fiber length is large enough (greater than 100–120 µm). Under this condition, the falling parts of force–crack length curves for the real specimen and the “equivalent cylinder”, including the *F*_max_ and *F_b_* values, are close to each other, and the equivalent cylinder can be used instead the real specimen shape.

For short embedded length, all seven methods underestimate the *τ_d_* value, but the alternative method yields the least error, since the difference in *F*_max_ values for the real specimen and the equivalent cylinder is smaller than the difference in *F_d_*. On the contrary, the traditional method based on the debond force results in the greatest *τ_d_* underestimation.

For some specimens, the force–displacement curve can include two kinks, and one of them may be due to crack propagation in the glue at the opposite fiber end. These kinks can be identified by comparing the *τ_d_* values obtained using the traditional and alternative methods. The *F_d_* value which shows better agreement between the two methods, corresponds to the “correct” kink.

## Figures and Tables

**Table 1:** Fiber and matrix properties and specimen dimensions.

Property	GF ^a^	CF1 ^b^	CF2 ^c^	PVA ^d^	Epoxy ^e^	PP ^f^	PA 6,6 ^g^	Concrete ^h^
Fiber diameter, *d_f_* (µm)	10–25	6–8	3–6	25–49	-	-	-	-
Radius of the matrix droplet, *R_m_* (mm)	-	-	-	-	1.25	1.25	1.25	1.3
Axial tensile modulus, *E_A_* or *E_m_* (GPa)	75	240	205	35	2.9	1.4	3.2	28
Axial Poisson ratio, *ν_A_*	0.17	0.2	0.2	0.2	0.35	0.35	0.3	n/a
Axial CTE, *α_A_* or *α_m_* (10^−6^ K^−1^)	5	−0.1	−0.9	n/a ^i^	76	150 ^i^	81	n/a
Stress-free temperature, *T_ref_* (°C)	-	-	-	-	80	23 ^i^	65	23 ^i^
Embedded length, *l_e_* (µm)	100–900	80–200	30–120	300–2000	-	-	-	-

^a^ Leibniz-Institut für Polymerforschung, Dresden, Germany; ^b^ Toho Tenax, Japan; ^c^ Sigrafil C, SGL Carbon Fibers Ltd., Wiesbaden, Germany; ^d^ Kuralon K-II REC15, Kuraray Co., Ltd. (Tokyo, Japan); ^e^ DGEBA-based epoxy matrix by Momentive Specialty Chemicals Inc., Columbus, OH, USA; ^f^ HG455FB polypropylene homopolymer by Borealis AG, Vienna, Austria; ^g^ Ultramid A27 PA 6,6 by BASF; ^h^ Leibniz-Institut für Polymerforschung, Dresden, Germany (see [58] for details); ^i^ If the stress-free temperature for a given fiber–matrix pair is equal to the test temperature, the coefficients of thermal expansion are not required for data evaluation.

**Table 2:** Interfacial strength parameters determined from a theoretical force–displacement curve for E-glass fiber–epoxy matrix pair using different methods.

Method	*F_d_*, N	*F*_max_, N	*F_b_*, N	*τ_d_*, MPa	*τ_f_*, MPa	*s* × 10^3^, N^2^	Rank
1 (*F_d_*, *F*_max_)	0.2939	0.4466	0.2284	53.53	7.27	5.11	7
2 (*F_d_*, *F_b_*)	0.2939	0.3982	0.1569	53.53	5.00	2.34	5
3 (*F_d_*, best{*F*_max_, *F_b_*})	0.2939	0.4132	0.1794	53.53	5.71	1.62	3
4 (*F_b_*, *F*_max_)	0.3472	0.4466	0.1569	60.90	5.00	2.84	6
5 (*F_b_*, best{*F_d_*, *F*_max_})	0.3180	0.4200	0.1569	56.87	5.00	1.29	2
6 (*F*_max_, best{*F_d_*, *F_b_*})	0.3288	0.4466	0.1827	58.35	5.81	1.88	4
7 (best { *F_d_*, *F*_max_, *F_b_*})	0.3133	0.4250	0.1712	56.21	5.45	1.04	1
Equivalent cylinder	0.3401	0.4412	0.1569	60	5	-	-
30° meniscus	0.2939	0.4466	0.1569	60	5	-	-

The embedded length is 500 µm, the fiber diameter is 20 µm, and the nominal (preset) strength parameters are *τ_d_* = 60 MPa and *τ_f_* = 5 MPa.

**Table 3:** Interfacial strength parameters determined from a theoretical force–displacement curve for E-glass fiber–epoxy matrix pair using different methods.

Method	*F_d_*, N	*F*_max_, N	*F_b_*, N	*τ_d_*, MPa	*τ_f_*, MPa	*s* × 10^3^, N^2^	Rank
1 (*F_d_*, *F*_max_)	0.05681	0.1181	0.07463	23.76	23.76	3.47	3
2 (*F_d_*, *F_b_*)	0.05681	0.05681	0.01572	23.76	5.00	3.76	4–7
3 (*F_d_*, best{*F*_max_, *F_b_*})	0.05681	0.05681	0.01572	23.76	5.00	3.76	4–7
4 (*F_b_*, *F*_max_)	0.1181	0.1181	0.01572	45.01	5.00	3.76	4–7
5 (*F_b_*, best{*F_d_*, *F*_max_})	0.08746	0.08746	0.01572	34.38	5.00	1.88	1–2
6 (*F*_max_, best{*F_d_*, *F_b_*})	0.1181	0.1181	0.01571	45.01	5.00	3.76	3
7 (best { *F_d_*, *F*_max_, *F_b_*})	0.08745	0.08745	0.01571	34.38	5.00	1.88	1–2
Equivalent cylinder	0.1730	0.1730	0.01572	60	5	-	-
30° meniscus	0.05681	0.1181	0.01572	60	5	-	-

The embedded length is 50 µm, the fiber diameter is 20 µm, and the nominal (preset) strength parameters are *τ_d_* = 60 MPa and *τ_f_* = 5 MPa.

**Table 4:** Interfacial strength parameters determined from the experimental force–displacement curve for E-glass fiber–PP matrix pair using different methods.

Method	*F_d_*, N	*F*_max_, N	*F_b_*, N	*τ_d_*, MPa	*τ_f_*, MPa	*s* × 10^3^, N^2^	Rank
1 (*F_d_*, *F*_max_)	0.1259	0.3296	0.3038	15.22	8.73	0.292	6
	0.2659	0.3296	0.1356	32.14	3.90	22.82	7
2 (*F_d_*, *F_b_*)	0.1259	0.3158	0.2867	15.22	8.24	0.191	5
	0.2659	0.4272	0.2867	32.14	8.24	9.53	5
3 (*F_d_*, best{*F*_max_, *F_b_*})	0.1259	0.3212	0.2935	15.22	8.44	0.116	2
	0.2659	0.3968	0.2419	32.14	6.95	6.53	3
4 (*F_b_*, *F*_max_)	0.1455	0.3296	0.2867	17.59	8.24	0.384	7
	0.1455	0.3296	0.2867	17.59	8.24	14.50	6
5 (*F_b_*, best{*F_d_*, *F*_max_})	0.1324	0.3203	0.2867	16.01	8.24	0.130	3
	0.2183	0.2869	0.2867	26.39	8.24	5.55	2
6 (*F*_max_, best{*F_d_*, *F_b_*})	0.1344	0.3296	0.2966	16.25	8.53	0.172	4
	0.1977	0.3296	0.2304	23.89	6.62	7.83	4
7 (best { *F_d_*, *F*_max_, *F_b_*})	0.1305	0.3230	0.2918	15.77	8.39	0.091	1
	0.2282	0.3736	0.2560	27.59	7.36	4.30	1
Experimental values	0.1259	0.3296	0.2867	-	-	-	-
	0.2659						

The embedded length was 629 µm, the fiber diameter was 17.6 µm. The upper value was calculated for the first kink (*F_d_*_1_ = 0.1259 N); the lower, for the second kink (*F_d_*_2_ = 0.2659 N).

**Table 5:** Interfacial strength parameters determined from the experimental force–displacement curve for PVA fiber–concrete matrix pair using different methods.

Method	*F_d_*, N	*F*_max_, N	*F_b_*, N	*τ_d_*, MPa	*τ_f_*, MPa	*s* × 10^3^, N^2^	Rank
1 (*F_d_*, *F*_max_)	0.2000	0.2924	0.1006	36.10	0.44	2.85	7
	0.2812	0.2924	0.0126	50.76	0.054	19.99	7
2 (*F_d_*, *F_b_*)	0.2000	0.3422	0.1540	36.10	0.67	2.48	5
	0.2812	0.4228	0.1540	50.76	0.67	16.99	5
3 (*F_d_*, best{*F*_max_, *F_b_*})	0.2000	0.3190	0.1292	36.10	0.56	1.32	3
	0.2812	0.3625	0.0891	50.76	0.39	9.13	3
4 (*F_b_*, *F*_max_)	0.1497	0.2924	0.1540	27.02	0.67	2.53	6
	0.1497	0.2924	0.1540	27.02	0.67	17.30	6
5 (*F_b_*, best{*F_d_*, *F*_max_})	0.1751	0.3175	0.1540	31.61	0.67	1.25	2
	0.2161	0.3581	0.1540	39.00	0.67	8.56	2
6 (*F*_max_, best{*F_d_*, *F_b_*})	0.1734	0.2924	0.1289	31.30	0.56	1.34	4
	0.2112	0.2924	0.0886	38.13	0.39	9.17	4
7 (best {*F_d_*, *F*_max_, *F_b_*})	0.1825	0.3100	0.1381	32.95	0.60	0.87	1
	0.2363	0.3376	0.1104	42.65	0.48	5.96	1
Experimental values	0.2000	0.2924	0.1540	-	-	-	-
	0.2812						

The embedded length was 1921 µm, the fiber diameter was 38.13 µm. The upper value was calculated for the first kink (*F_d_*_1_ = 0.200 N); the lower, for the second kink (*F_d_*_2_ = 0.2812 N).

**Table 6:** Interfacial strength parameters determined from the experimental force–displacement curve for carbon fiber (CF2)–H135 epoxy matrix pair using different methods.

Method	*F_d_*, N	*F*_max_, N	*F_b_*, N	*τ_d_*, MPa	*τ_f_*, MPa	*s* × 10^3^, N^2^	Rank
1 (*F_d_*, *F*_max_)	0.05197	0.06386	0.06181	89.25	75.92	2.935	7
2 (*F_d_*, *F_b_*)	0.05197	0.51973	0.00764	89.25	9.38	0.141	3–6
3 (*F_d_*, best{*F*_max_, *F_b_*})	0.05197	0.05197	0.00764	89.25	9.38	0.141	3–6
4 (*F_b_*, *F*_max_)	0.06386	0.06386	0.00764	107.47	9.38	0.141	3–6
5 (*F_b_*, best{*F_d_*, *F*_max_})	0.05792	0.05792	0.00764	98.36	9.38	0.071	1–2
6 (*F*_max_, best{*F_d_*, *F_b_*})	0.06386	0.06386	0.00766	107.47	9.41	0.141	3–6
7 (best { *F_d_*, *F*_max_, *F_b_*})	0.05792	0.05792	0.00764	98.36	9.38	0.071	1–2
Experimental values	0.05197	0.06386	0.00764	-	-	-	-

The embedded length was 47 µm, the fiber diameter was 5.5 µm.
